# Fabrication of MoO_3_ Nanowire-Based Membrane Devices for the Selective Adsorption of Cationic Dyes from Aqueous Solutions with High Performance and Reusability

**DOI:** 10.3390/mi10090586

**Published:** 2019-08-31

**Authors:** Yifan Zhang, Soo-Jin Park

**Affiliations:** Department of Chemistry and Chemical Engineering, Inha University, 100 Inharo, Incheon 22212, Korea

**Keywords:** MoO_3_, nanowires, cationic dyes, repeatability

## Abstract

A series of ultralong (up to tens of micrometers) MoO_3_ nanowire-based membranes were synthesized for the treatment of aqueous solutions containing the cationic dyes methylene blue (MB) and rhodamine B (RhB). This treatment method possesses extremely rapid and superhigh adsorbability (up to 521 and 321 mg/g for MB and RhB, respectively), as well excellent selective adsorption ability of cationic dyes with respect to the anionic dye methyl orange (MO). Moreover, the cationic dyes on the membrane can be desorbed easily, and reusability is good.

## 1. Introduction

Recently, water contamination by dyes has been attracting much attention. Among the contaminating dyes, cationic dyes, such as methylene blue (MB) and rhodamine B (RhB), are widely used in dying cotton, printing, and dying textiles [1,2,3]. Although MB and RhB are not regarded as very toxic dyes, they also can produce some harmful effects to human health. Consequently, industrial effluent containing MB and RhB has to be treated before discharge. Recently, various methods have been developed to solve this issue, such as photocatalytic degradation, flocculation–coagulation, precipitation, and filtration [4,5,6,7,8,9,10]. Comparatively speaking, membrane filtration has received more attention because of its low cost, high efficiency, and flexible design. To date, although many nanomaterials including activated carbon, carbon nanotube, and graphene [11,12,13] have been explored to remove dyes from wastewater, there are still some disadvantages in their use that need to be overcome, such as high cost, unrecyclability, and possible secondary pollution [14,15,16,17,18]. Therefore, it is necessary to develop desirable materials that not only can adsorb dyes with high efficiency but also can be reused with good recyclability. Molybdenum oxide (MoO_3_), a prominent material among metal oxides, has been regarded as a suitable choice owing to its chemical stability, nontoxicity, high adsorbability, and low price [19,20,21,22,23]. Up till now, MoO_3_ has been used in various applications including photocatalysis, supercapacitors, photovoltaics, batteries, and electrocatalysis [24,25,26,27]. However, research about dye adsorption by MoO_3_ nanowire-based membranes is still limited. With this in mind, we designed and synthesized a type of MoO_3_ nanowire-based membrane to investigate its adsorption properties and recyclability behavior for dye removal.

## 2. Experimental Section

In this research, we synthesized a novel MoO_3_ nanowire-based membrane at various thicknesses. The MoO_3_ nanowires could be prepared by a hydrothermal method. The procedure is explained in detail in the Electronic Supporting Information. This kind of nanowire could easily be assembled into a flexible film by a vacuum and pressing procedure. This MoO_3_ nanowire-based membrane exhibited a superhigh adsorbability, as well as excellent selective adsorption of cationic dyes (Figure 2 and Figure 3).

## 3. Results and Discussion

**Figure 1:**
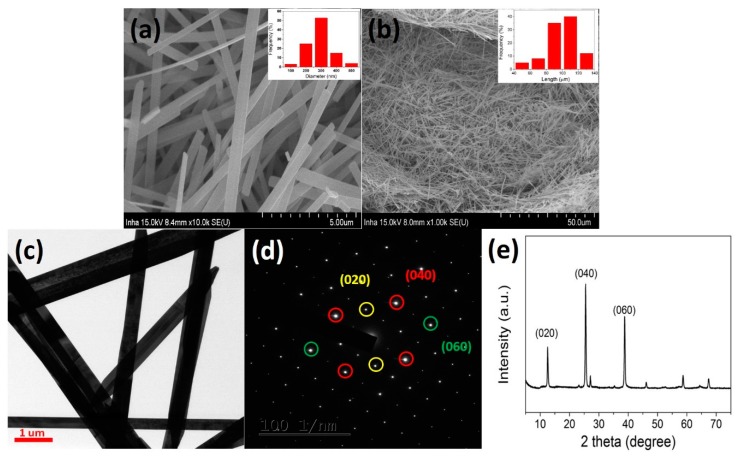
(**a**) Scanning electron microscope (SEM) of molybdenum oxide (MoO_3_) nanowires at high resolution (diameter distribution inset); (**b**) SEM of MoO_3_ nanowires at low resolution (length distribution inset); (**c**) Transmission electron microscope (TEM) of MoO_3_ nanowires; (**d**) corresponding selected-area electron diffraction (SAED) pattern; (**e**) X-ray diffraction (XRD) patterns of MoO_3_ nanowires.

The surface morphology of the as-synthesized nanowires is shown in Figure 1a,b. All the as-prepared samples showed the presence of wire-like MoO_3_ with a diameter of 200−400 nm and a length of 80–100 µm. Besides, the transmission electron microscope (TEM) observations and the phase information were confirmed by selected-area electron diffraction (SAED), as displayed in Figure 1c and d. The SAED patterns exhibited disparate diffraction spots, revealing the hybrid characteristics of MoO_3_. Meanwhile, X-ray diffraction (XRD) was also used to clarify the phase purities and crystal structures of the as-synthesized samples. The diffraction peaks at 12.6°, 25.4°, and 38.8° could be indexed to the (020), (040), and (060) planes of MoO_3_ (PDF No. 05-0508) in Figure 1e, respectively, which indicated high phase purity [28,29,30]. As mentioned above, the as-prepared membrane was composed of nanowires with a diameter of 400 nm and a length of hundreds of micrometers. We found that it was extremely stable in aqueous solution without sediment even after one day (Figure S1). As we know, after the vacuum-driven process, a large number of pores with various diameters form and are effective for molecular filtration.

**Figure 2:**
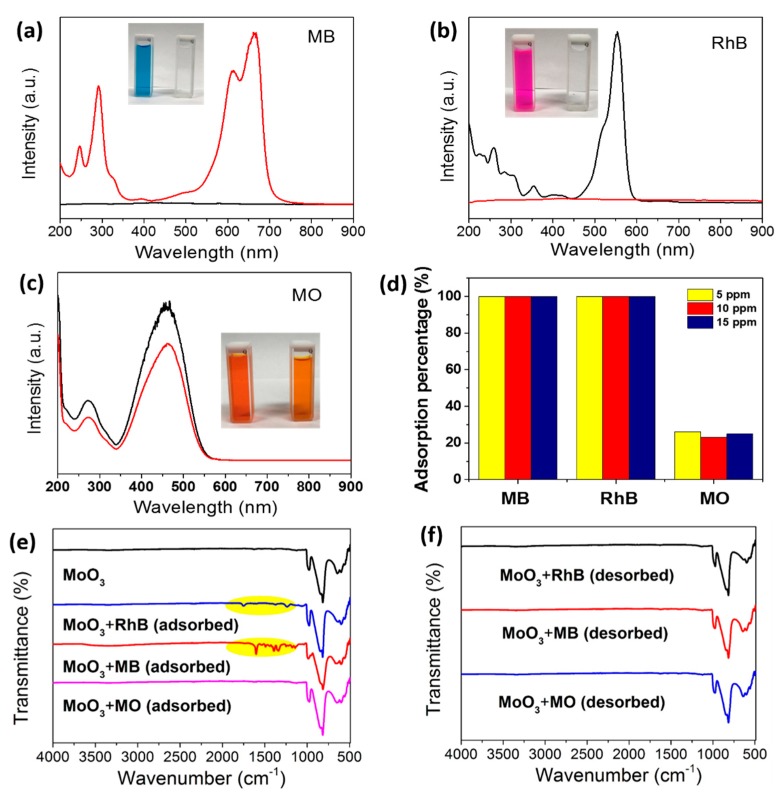
The prepared MoO_3_-based membrane (5 μm) was used to vacuum-filtrate the dyes at different concentrations (ppm): (**a**) 10 ppm of methylene blue (MB); (**b**) 10 ppm of rhodamine B (RhB); (**c**) 10 ppm of methyl orange (MO); (**d**) adsorption percentage in ppm for different dyes; (**e**) FT-IR spectrum of the membrane: dyes adsorbed; (**f**) FT-IR spectrum of the membrane: dyes desorbed.

The separation performance of the membrane (5 μm) for different dyes is shown in Figure 2. The UV–vis spectra of the aqueous dye solution (10 ppm) before and after filtration are displayed in Figure 2a–c. It is clear that after vacuum filtration, MB and RhB were totally removed (see also Movie S1 and Movie S2 in the Supporting Information). Meanwhile, only 24.6% of MO was removed under the same conditions, which is in agreement with the phenomenon shown in Movie S3. The FT-IR spectra of the adsorbed and desorbed membranes are displayed in Figure 2e,f, in which more peaks appear between 1200 and 1700 cm^−1^, which can be ascribed to the adsorbed dyes on the surface. After desorbing, the peaks between 1200 and 1700 cm^−1^ disappeared. This confirmed that the dyes could be adsorbed and desorbed easily in the presence of MoO_3_ nanowires. The membranes after filtration are shown in Figure S2. The front and back sides of the membranes indicate that there were no MB and RhB on the back side, which is in agreement with Figure 2. Moreover, we obtained the same results when increasing the ppm level (Figure 2d). In addition, the permeation performance of membranes with different thicknesses and various pH values was evaluated by examining the dye aqueous solution fluxes through the membranes. As presented in Figure S3a, the flux curve was consistent with the Hagen–Poiseuille theoretical models (Equation (1)) [31].

(1) $$\Delta P=\frac{8\mu LQ}{\pi R^{4}}$$

where Δ*P* is the pressure difference between the two ends, *L* is the length of pipe, *μ* is the dynamic viscosity, *Q* is the volumetric flow rate, and *R* is the pipe radius.

The water flux of the 5 μm membrane was 3782 L·h^−1^·m^−2^·bar^−1^, which is higher than that of traditional commercial filtration membranes (Figure S3a). The dramatically enhanced permeation performance is believed to be due to the pores in the membrane, which provide unobstructed channels for the water to pass, and to the ultrathin membrane thickness, which offers a low friction resistance. We also measured the separation performance at various pH values (Figure S3b), MB solutions at various pH values were prepared using 1 mol/L of HCl or NaOH. The results indicate that the as-prepared membranes had excellent pH tolerance and, therefore, an excellent application prospect.

**Figure 3:**
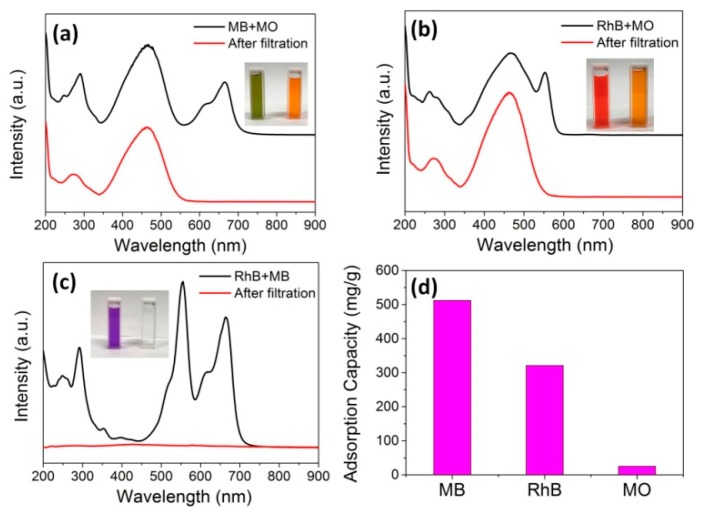
The MoO_3_-based membrane (5 μm) was tested to measure its ability to selectively separate various dyes at 10 ppm: (**a**) MB + MO; (**b**) RhB + MO; (**c**) MB + RhB; (**d**) Adsorption capacity for various dyes.

**Scheme 1:**
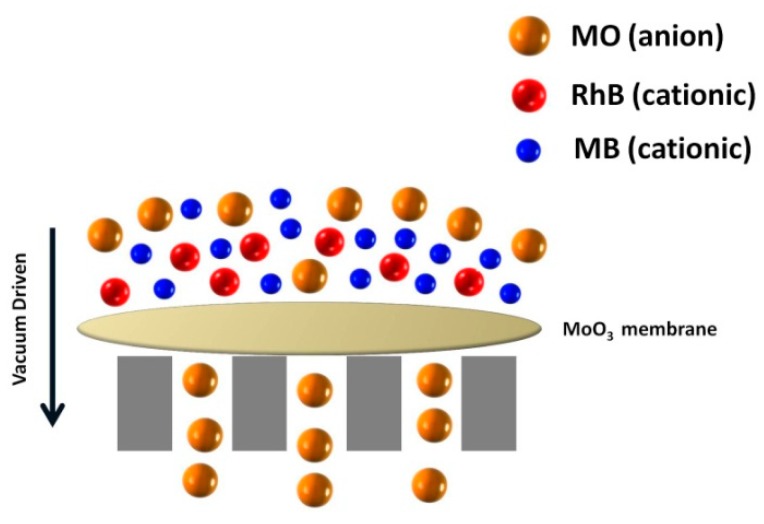
Process of selective adsorption of cationic dyes from blended dyes.

We further investigated the selective adsorption performance of the membrane during filtration. Dye mixtures of MB +MO, RhB + MO, MB + RhB were prepared (10 ppm). For comparison, the UV–vis spectra of dye mixtures were measured, and the spectra are shown in Figure 3a–c (black lines). We also collected the filtered solutions, whose spectra are also displayed in Figure 3a,b (red lines). As shown in Movie S4 (MB + MO) and Movie S5 (RhB + MO), when the dye mixture aqueous solution was filtered through the MoO_3_ membrane, the filtered solution turned orange for MB + MO and RhB + MO. Regarding the mixture MB + RhB, the solution became transparent. These results proved the selective adsorption performance for MB and RhB. The adsorption capacity is another crucial parameter for practical applications. Figure 3d shows adsorption capacities of 512 mg/g (MB), 321 mg/g (RhB), and 25 mg/g (MO). The adsorption capacities of MB and RhB were almost 20 and 13 times higher than that for MO), which is in agreement with the basic theory underlying the selective adsorption performance for cationic dyes. Furthermore, the adsorption capacity of the membrane was much higher than that of commercial filtration adsorbents (Table S1), such as activated carbon, transition metal oxides, and other biomass-derived carbon adsorbents [32,33,34]. The process of selective adsorption of cationic dyes is shown in Scheme 1.

**Figure 4:**
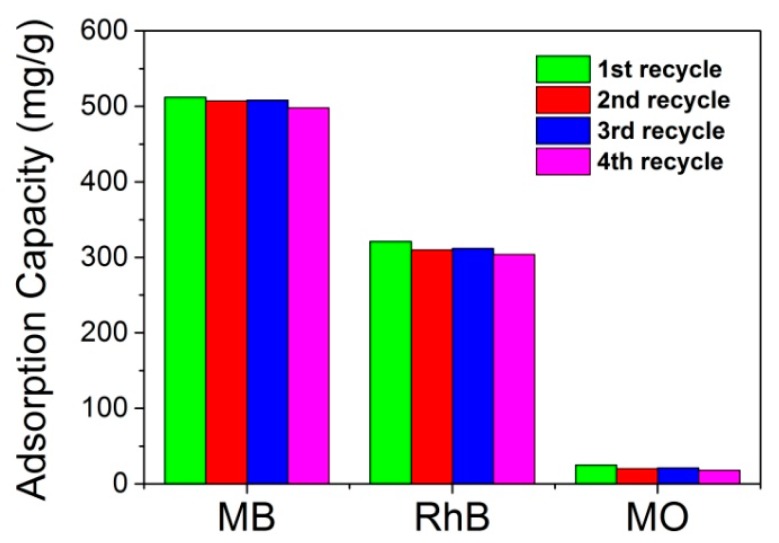
Reusability of the MoO_3_ nanowire-based membrane for dye (MB, RhB, MO) adsorption.

Apart from the fast adsorption rate and high adsorption capacity, a high reusability is equally important for a good adsorbent. In our study, we also conducted a reusability test under the same conditions. The regeneration process of the MoO_3_ nanowire-based membrane is displayed in the Supporting Information. As described, the used MoO_3_ nanowire-based membrane (0.5 g) was redispersed in ultrapure water by sonication and then activated with a dilute solution of NaCl (100 mL, 2 mol/L) and dimethylformamide (DMF) (50 mL) under sonication at room temperature to remove the adsorbed MB, RhB, or MO. The re-generated MoO_3_ nanowires were washed with water and ethanol several times until they became neutral and reused for the next adsorption experiment. For the reusability study, 0.5 g of as-prepared MoO_3_ nanowires were re-dispersed into 100 mL of deionized water and sonicated for 30 min to form a milky suspension. Then, the suspension was slowly transferred onto a membrane filter (Polytetrafluoroethylene, 0.45 μm) under a vacuum pressure of 0.1 MPa. After drying and peeling off the supporting substrate, the MoO_3_ nanowire-based membrane was ready to be reused in the same process. The same procedure was conducted four times, and the results were shown in Figure 4. After each recycling step, the regenerated MoO_3_ nanowires were added into the dye aqueous solutions (10 ppm). It is obvious that the adsorbent was still able to remove more than 90% of each dye after four cycles. Furthermore, it is known that the wettability of materials is a crucial factor affecting their filtrating performance. As shown in Figure S4, the contact angle of the membrane was 111.48, and that of MoO_3_ is 34.12 degree. Therefore, the MoO_3_ nanowire-based membrane is hydrophilic, and this characteristic facilitates water transfer among materials during the filtration process. These inherent characteristics are of great value for practical use. Meanwhile, we also try to compare the adsorption ability with those of other adsorbents reported in the literature. Detailed information is shown in Table 1.

## 4. Conclusions

In conclusion, we have developed a facile method to synthesize a MoO_3_ nanowire-based membrane, which was used for the selective adsorption of cationic dyes from aqueous solutions of mixed dyes. The adsorption rate and adsorption capacities of the new membrane are higher than those of traditional commercial membranes. Notably, the recyclability of this material is outstanding, as its adsorption ability is maintained after four cycles. Moreover, a limited loss of performance during the recycling procedure can reduce the costs, and suggests a bright future for the practical application of this system. Lastly, we hope this system can be of inspiration for the design of similar materials for the selective separation of other organics with low molecular weight.

## Figures and Tables

**Table 1:** Maximum adsorption abilities of MB for various adsorbents.

Adsorbent.	pH	Temp/K	q_max_ (mg/g)	References
Activated carbon	7.0	293	91.0	[35]
Graphene	3.0	293	153.85	[36]
Graphene Oxide	6.0	298	243.9	[37]
Carbon nanotubes	7.0	298	46.2	[38]
Cotton stalk	7.0	308	147.06	[39]
Chitosan/graphene oxide	5.3	303	95.16	[40]
MoO_3_ nanowires	7.0	298	521.0	This study

## Supplementary Materials

The following are available online at https://www.mdpi.com/2072-666X/10/9/586/s1, Figure S1: (a) As prepared MoO_3_ nanowires sonicated for 30 min in the water; (b) Standing for one day, Figure S2: Front side and back side of as-synthesized MoO_3_ nanowires membrane after filtrating with various dyes, Figure S3: (a) Variation of MB solution flux with different membrane thickness; (b) Adsorption percentage of 5 μm membrane under different pH, Figure S4: Contact angle tendency of as-prepared MoO_3_ nanowires based membrane (34.12°), Table S1: Comparison of MB adsorption capacity of the as prepared MoO_3_ nanowires with other reported adsorbents, Video S1: Methylene blue (MB), Video S2: Rhodamine B (RhB), Video S3: Methyl orange (MO), Video S4: Methylene blue (MB) + Methyl orange (MO), Video S5: Rhodamine B (RhB) + Methyl orange (MO).
